# HIV-2 neutralization by intact V3-specific Fab fragments

**DOI:** 10.1186/1743-422X-5-96

**Published:** 2008-08-18

**Authors:** Samer Sourial, Charlotta Nilsson

**Affiliations:** 1 Department of Microbiology, Tumor and Cellbiology, Karolinska Institute, Stockholm, Sweden; 2 Department of Immunology and Vaccinology, Swedish Institute for Infectious Disease Control, Solna, Sweden; 3 AstraZeneca Safety Assessment, Södertälje, Sweden

## Abstract

The V3 region of both HIV-1 gp120 and HIV-2 gp125 surface glycoprotein has been described as a target for neutralizing antibodies. In this study a conformation-sensitive (3C4) and a linear site-specific (7C8) anti-HIV-2 V3 monoclonal antibody (mAb) were characterized. The neutralization capacity of the purified mAbs and their respective papain-generated Fab fragments was analyzed. The Fabs were further characterized by sequence analysis. Our results demonstrate that neither purified mAbs were capable of neutralizing HIV-2, while intact Fab fragments from both mAbs blocked in vitro infection of HIV-2 isolates. Moreover, the conformation sensitive 3C4 Fab neutralized both subtype A and B HIV-2 isolates and SIVsm. Sequence analysis of the hypervariable regions of 3C4 Fab and 7C8 Fab revealed that the third CDR of the heavy chain (CDRH3) of the antibodies was not as long as many of the previously characterized neutralizing antibodies. Our findings suggest that whole 7C8 and 3C4 mAbs are sterically hindered from neutralizing HIV-2, whereas the smaller size of Fab fragments enables access to the V3 region on the virion surface.

## Findings

The HIV-1 V3 region has been identified as a target site recognized by neutralizing antibodies [1]. Monoclonal antibodies (mAbs) targeting conformational epitopes within the V3 region have been demonstrated to neutralize primary HIV-1 isolates [2-5]. Furthermore, anti-HIV-1 V3-specific mAbs have recently been shown to have broad cross-reactivity, which was dependent on the extent of masking of the V1/V2 regions and the sequence at the crown of the V3-loop [6].

Neutralizing antibodies have been reported to bind within the HIV-2 V3 region [7,8], with the FHSQ residues at the tip of the V3-loop [9]. However, mAbs recognizing the linear (FHSQ) site on gp125 could not neutralize HIV-2 isolates [9,10]. Conversely, a conformational epitope composed of FHSQ (amino acids 315–318 in HIV-2 ISY clone) and WCR (amino acids 329–331) has been described in the V3 region of gp125 [9,11], where mAbs recognizing conformational epitopes in the gp125 V3 region could neutralize HIV-2 isolates [9,12]. While the exposure of the V3 region in HIV-1 is debated, the accessibility of neutralizing sites on HIV-2 V3 region has not been as extensively characterized as for HIV-1.

Previously, two hybridoma cell-lines have been isolated from mice immunized with two overlapping peptides of HIV-2 spanning the center and C-terminus of the V3 region [9]. The hybridoma expressing the 3C4 mAb recognized both peptides, while the 7C8 mAb recognized only the center of the V3 region. Mouse ascitic fluid containing 3C4 has been reported to neutralize different isolates of HIV-2 at a dilution of 1:20, whereas the mouse ascitic fluid containing 7C8 had no neutralizing effect. Further characterization of the binding of these two mAbs to recombinant gp125 indicated that 3C4 is conformation sensitive while 7C8 binds to a linear site [13].

In this study, the HIV-2 neutralization capacity of protein A-purified 3C4 and 7C8 mAbs was analyzed. A neutralization assay employing phytohemagglutinin-stimulated PBMCs (peripheral blood mononuclear cells) was used [14]. Two-fold serial dilutions of mAb starting at 100 μg/ml were incubated for one hour at 37°C with a minimum of 15 TCID_50 _(50% tissue culture infectious dose) tissue culture supernatant from virus infected cells. PBMCs (10^5^) were then added to the mix and incubated overnight at 37°C. The medium was changed with fresh IL-2 containing medium on the following day and on day 4. Seven days after infection, supernatants were collected and analyzed for HIV-2 antigen by a capture ELISA [15]. The neutralization concentration was defined as the concentration where a 80% reduction or more of optical density at 490 nm in the culture supernatant was seen as compare to the negative control (i.e. IC_80_). To determine the virus inoculum dose, a TCID_50 _titration was performed in parallel to each neutralization experiment.

Contrary to what has been reported previously for 3C4, up to 100 μg/ml of the purified mAbs did not neutralize any of the HIV-2 isolates tested (data not shown). This could be explained by the fact that ascitic fluid contains > 10 mg/ml of mAb as well as other components that could alter the neutralization effect.

The lack of neutralization capacity displayed by both the linear-specific (7C8) and conformation-sensitive (3C4) antibodies may be related to the accessibility of the epitope recognized by these antibodies. Previous reports on the potent neutralization effect of Fabs compared to mAbs [16,17] prompted us to study the effect of the size of mAbs on HIV-2 neutralization capacity. Both 7C8 and 3C4 were digested using papain, and the different digestion products were separated using size-exclusion chromatography (Amersham Biosciences). Fab fragments were purified using Superose-12 and eluted in a single peak using 20 mM Tris buffer. Fractions from this peak were pooled and used in the neutralization assays. HIV-2_SBL6669 _is a CXCR4 co-receptor-using isolate belonging to subtype A. In contrast to the undigested mAbs, both 3C4 and 7C8 Fabs were capable of neutralizing the homologous HIV-2_SBL6669 _isolate (including the V3 sequence the mAbs were generated against) and the hetrologous CCR5 co-receptor-using laboratory-adapted isolate HIV-2_K135_. Table 1 indicates the concentrations of 3C4 and 7C8 Fabs required to neutralize HIV-2 as determined on at least two different occasions. HIV-2_SBL6669 _and HIV-2_K135 _could be neutralized by both 7C8 and 3C4 Fabs, where 3C4 Fab was capable of viral neutralization at as low a concentration as 0.16 μg/ml. The concentration of 3C4 Fab required for neutralization was dependent on the virus inoculum dose, whereas 0.63 μg/ml of 7C8 Fab was required in the cases where neutralization was observed. In all the neutralization assays, the 3C4 Fab appeared to be twice as potent in neutralization as was the 7C8 Fab. Furthermore, all the primary isolates tested, regardless of co-receptor usage, were neutralized by the 3C4 Fab. Since the neutralization sites recognized by 3C4 are conserved in both HIV-2 and SIV, the neutralization of SIV_sm _was also assessed. SIV_sm _was as efficiently neutralized by the 3C4 Fab as the other HIV-2 isolates tested.

**Table 1:** *In vitro *virus neutralization exhibited by 7C8 Fab and 3C4 Fab fragments given as the concentration of Fab needed for IC_80 _neutralization.

	**Virus**			**Fab Fragment**		
		
Isolate	Subtype	Coreceptor usage^+^	TCID_50_		7C8 (μg/ml)	3C4 (μg/ml)
		
HIV-2_SBL6669_	A	CCR3, CXCR4	42		0.63	0.31
			15		0.63	< 0.16
HIV-2_K135_	unknown	CCR5*	435^		-	6.25
			18		0.63	0.31
HIV-2_1682_	A	CCR1, CCR3, CCR5	28		-	6.25
HIV-2_1653_	B	CCR5	18		-	6.25
HIV-2_2298_	A	CCR5	18		-	12.50
SIV_sm_	n/a	CCR5, BOB	44		-	3.13

^+ ^Co-receptor usage according to Mörner et al. [11] and Vödrös et al. [30].

* Determined by Jose M. Marcelino (personal communication).

n/a Not applicable.

^ Very high virus inoculum dose.

- No neutralization observed up to 100 μg/ml.

**Figure 1:**
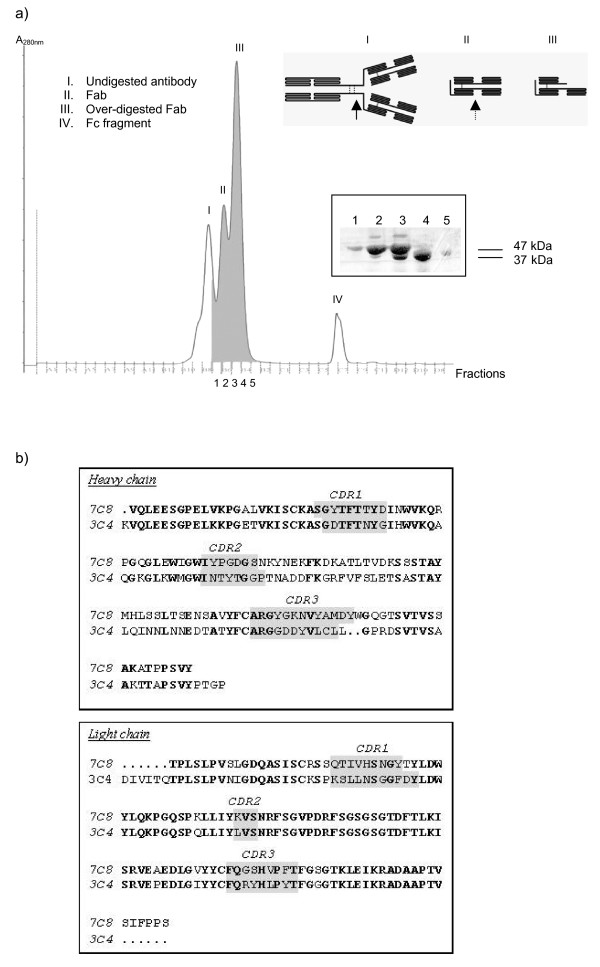
**Fab production, purification and sequencing**. *A*. Chromatogram of separation of papain digestion products using Superdex-75. The undigested mAb (I) is eluted first, followed by the Fab fragments (II & III) and the Fc fragments along with other small digestion products (IV) are collected last. *Bottom right: *Analysis of fractions 1–5 on 10% SDS gel demonstrate separation of the Fab fragments (47 kDa) from the over-digested Fab fragments (≈ 37 kDa). *Top right: *Schematic diagram of mAb digestion by papain as previously described in literature [18]. The bold arrow indicates were papain digests primarily before the cysteine-bridges on mAb (I). The papain can also digest the Fab fragment produced (II), as indicated by dashed arrow. The digested Fab (III) may be missing one of the Fv regions. *B*. The variable regions of 7C8 and 3C4 heavy and light chains, are aligned respectively. The CDR regions as indicated by immunoglobulin superfamily database [31] are shaded.

The level of neutralization varied depending on the quality of the Fab produced by the papain digestion, where the digestion efficiency varied between experiments. To further analyze the variation in neutralization efficiency of the generated Fab fragments, Superdex 75 size-exclusion chromatography column was used. Figure 1A depicts the separation of digestion products, where Fabs (peak II) are separated from over-digested Fab fragments (peak III). Fab fragments of molecular weight ~47 kDa were capable of neutralizing HIV-2, whereas over-digested Fab fragments with a lower molecular weight of ~37 kDa were not (data not shown). The preferential papain digestion sites on antibodies is illustrated in the schematic diagram in Figure 1A. As previously described, over-digestion by papain can release Fv from Fab fragments [18,19], producing a digested Fab fragment incapable of viral neutralization.

The mechanism underlying the more potent neutralizing effect of Fab fragments has been previously correlated with the smaller size of Fab fragments compared to the size of whole IgG, where steric factors could limit the accessibility to neutralizing epitopes [16]. Previous reports have also described different neutralization mechanisms between mAbs and Fabs at the attachment or fusion of the virus with target cells [20,21].

To characterize the Fab fragments, the variable regions of 7C8 and 3C4 were sequenced according to their subtypes. Sub-typing of 7C8 and 3C4 revealed that both mAbs have kappa light chains and that they belong to subtypes IgG1 and IgG2a, respectively. Alignment of 7C8 and 3C4 variable regions (Figure 1B) indicated that the light chains of both Fabs are more conserved (93% identity) than the heavy chain (53% identity). The predicted CDR loops are indicated in Figure 1B, where the CDR3 loop of 7C8 and 3C4 heavy chains consists of 13 and 11 residues, respectively.

The third CDR of the heavy chain (CDRH3) of an antibody plays a distinctive role in determining antibody specificity [22]. Sequence analysis of 7C8 CDRH3 revealed a comparatively long region of 13 amino acids (aa), whereas the average length of CDRH3 in mice is between 8 and 9 aa and <11% of mice antibodies have a CDRH3 length of >13 aa [22,23]. Most human anti-gp120 antibodies have CDRH3 of >15 aa [24], as exemplified by the neutralizing mAbs/Fab, 17b, b12, 2F5, and X5 which recognize different epitopes on gp120 and have CDRH3 lengths of 19, 18, 22, and 22 aa respectively [25-29]. Therefore, we do not believe that the comparatively longer CDRH3 regions of 3C4 and 7C8 would play a strong role in the viral neutralization.

In conclusion, a confirmation sensitive HIV-2 V3 specific Fab was shown to neutralize both HIV-2 A and B subtypes and SIV_sm_. Further analyses indicate that the smaller size of intact Fab fragments may be a determining factor for HIV-2 V3-specfic viral neutralization. These results could provide clues to small molecule anti-HIV inhibitors.

## Competing interests

The authors declare that they have no competing interests.

## Authors' contributions

SS participated in the design of the study, performed the mAb and Fab preparations and their characterization, analysed the data and drafted the manuscript, CN participated in the design of the study, performed the neutralization assays, analysed the data and helped to draft the manuscript. Both authors have read and approved the final manuscript.
